# Attenuated afferent inhibition correlated with impaired gait performance in Parkinson’s disease patients with freezing of gait

**DOI:** 10.3389/fnagi.2024.1458005

**Published:** 2024-12-18

**Authors:** Puyuan Wen, Hong Zhu, Zaichao Liu, Amin Chang, Xianwen Chen

**Affiliations:** ^1^Department of Neurology, The First Affiliated Hospital of Anhui Medical University, Hefei, China; ^2^Department of Neurology, Affiliated Yantai Yuhuangding Hospital of Qingdao University, Yantai, China

**Keywords:** Parkinson’s disease, freezing of gait, short-latency afferent inhibition, long-latency afferent inhibition, paired associative stimulation, sensory-motor integration

## Abstract

**Background:**

The neural mechanisms underlying freezing of gait (FOG) in Parkinson’s disease (PD) have not been completely comprehended. Sensory-motor integration dysfunction was proposed as one of the contributing factors. Here, we investigated short-latency afferent inhibition (SAI) and long-latency afferent inhibition (LAI), and analyzed their association with gait performance in FOG PD patients, to further validate the role of sensorimotor integration in the occurrence of FOG in PD.

**Methods:**

Twenty-five levodopa responsive-FOG PD patients (LR-FOG), fifteen levodopa unresponsive-FOG PD patients (LUR-FOG), twenty-eight PD patients without FOG (NO-FOG PD) and twenty-two healthy controls (HC) were included in the study. Clinical features such as PD motor symptoms, FOG severity and cognitive abilities were evaluated using clinical scales in subjects with PD. All participants underwent paired associative stimulation (PAS) to evaluate SAI and LAI in addition to regular input-output curve by transcranial magnetic stimulation. The performances of gait were assessed using a portable gait analyzing system in 10-meter timed Up and Go task. The correlations between the gait spatiotemporal parameters or the scores of FOG scale and the magnitudes of SAI or LAI were analyzed.

**Results:**

Compared to HC and NO-FOG PD patients, SAI was decreased in FOG PD subgroups. LAI was also reduced in both LR-FOG PD and LUR-FOG PD in relative to HC; however, only LUR-FOG PD showed significant reduction of LAI in comparison to NO-FOG PD group. FOG PD patients showed poorer gait performance compared to HC and NO-FOG PD group. The reduction of SAI and LAI were correlated with the impaired gait spatiotemporal parameters or scores of FOG scale in PD with FOG.

**Conclusion:**

The SAI and LAI were attenuated in PD patients with FOG, and the reduction of SAI or LAI were correlated to disturbed gait performance, indicating that sensory-motor integration dysfunction played a role in the development of FOG in PD.

## 1 Introduction

Freezing of gait (FOG) is a gait disturbance characterized by recurrent, transient episodes of gait hesitation and arrest in advanced Parkinson’s disease (PD), and it is a frequent episodic phenomenon often leads to falls and fractures (Giladi, 2001; Lamberti et al., 1997). Moreover, most patients do not respond well to medication, and it’s unclear if deep brain stimulation works well for FOG. This condition becomes increasingly recognized as a major cause of disability in PD patients, drawing more attention from both clinical and scientific perspectives (Walton et al., 2015).

FOG can be classified into three subtypes based on the response to medication: levodopa-responsive FOG(LR-FOG) (responding to dopaminergic treatment, occurring only during the off-phase of medication), levodopa-unresponsive FOG(LUR-FOG) (ineffective response to dopaminergic drugs, occurring during both on-phase and off-phase of medication), and levodopa-induced type (induced by dopaminergic medication, occurring only during the on-phase of medication) (Factor et al., 2014).

The mechanism of FOG is still unclear. Some theories suggest that sensory-motor integration dysfunction contributes to the occurrence of FOG (Almeida et al., 2005; Tan et al., 2011). Patients with FOG exhibit reduced proprioceptive and kinesthetic integration abilities, and decreased perception of spatial vision may be one of the reasons for postural balance and gait disturbances (Tan et al., 2011). Research has shown that sensory input can exacerbate or alleviate FOG; FOG is often exacerbated when patients pass through narrow passages or obstacles, and reducing proprioceptive input can also worsen FOG (Cowie et al., 2010). Certain visual and auditory cues in the environment (such as rhythmic auditory information and ground stripes) can temporarily improve FOG (Morris et al., 1996; Jiang and Norman, 2006). These signs point to a potential role for dysfunctional sensory-motor integration in the mechanism of FOG.

Paired associative stimulation (PAS) is a research technique employed to the influence of peripheral sensory input on excitability of the motor cortex (Stefan et al., 2000; Castel-Lacanal et al., 2009). It can be used to assess the cortical sensorimotor integration function by observing the impact of peripheral sensory input on TMS-induced motor evoked potential (MEPs) amplitudes in the targeted muscle. Afferent inhibition refers to the attenuation of the muscle reaction caused by a preceding conditioning electrical stimulus to a peripheral nerve in relative to the unconditioned TMS and was used to assess inhibitory circuits in the sensorimotor cortex non-invasively (Tokimura et al., 2000; Chen et al., 2008). According to the inter-stimulus intervals between the electrical stimulation of the peripheral nerve and the single pulse of TMS on motor cortex, two types of afferent inhibition can be detected, namely, short-latency afferent inhibition (SAI, with an interval of 20∼50 ms) and long-latency afferent inhibition (LAI, with an interval of 100∼200 ms). The alterations of SAI in PD were inconsistent in published literature, with most studies reporting reduced SAI, especially in medicated PD patients. A study revealed that PD patients who have not received drug treatment showed no difference in SAI compared to controls, but SAI was reduced after dopamine treatment (Dubbioso et al., 2019). A PAS study (Sailer et al., 2003) found that LAI is impaired in PD patients and cannot be corrected by levodopa, suggesting its association with non-dopaminergic neurotransmitter mechanisms. The characteristics of afferent inhibition and the association of SAI or LAI with gait performance in PD patients with FOG are not well studied until recently and the findings from various authors were inconclusive. For instance, Picillo et al. (2015) discovered no anomalies of SAI in PD-FOG patients in contrast to PD without FOG or age-matched healthy controls (HC); however, Wang et al. (2022) found that the thalamocortical cholinergic-GABAergic SAI pathway related to FOG were impaired. In addition, given the heterogeneous of gait disorders in PD, which can be caused by various neurotransmitters and circuits, it is conceivable that maybe there is an intricate relationship between SAI/LAI and gait deficits; so far, there is a scarcity of study evaluating SAI or LAI in different subtypes of PD-FOG.

In the current study, afferent inhibition (including SAI and LAI) and gait performance were assessed in LUR-PD patients as well as LR-FOG patients, and correlations between alterations of SAI or LAI and gait performance were analyzed in order to further explore potential impact of cortical sensorimotor integration dysfunction on FOG in PD patients.

## 2 Materials and methods

### 2.1 Study population

We sequentially enrolled individuals who satisfied the clinical criteria of the Movement Disorder Society (MDS) for PD (Goetz et al., 2008). The following were the criteria used to include PD patients in this study: (1) with a disease duration of at least 3 years but less than 10 years; (2) to be able to walk independently for a distance of more than 30 meters; (3) have a stable and optimized daily dose of anti-Parkinsonian medicine during the 4 weeks leading up to enrollment. The exclusion criteria included: (1) contraindications for TMS include the presence of metallic objects within the skull; (2) interference from severe tremors or medication-induced motor fluctuations in the “on” state with electromyography (EMG) recordings; (3) significant anxiety and depression diagnosed in accordance with the Fourth Edition of the Diagnostic and Statistical Manual of Mental Disorders; (4) dementia diagnosed using MDS criteria (Emre et al., 2007); (5) use of anticholinergic or antidepressant medications; (6) other diseases that interfere with gait, such as ischemic stroke and normal-pressure hydrocephalus. The recruitment process occurred between March 2023 and February 2024, encompassing all eligible patients with PD at our center within a 1-year timeframe. In addition, patients with PD who have FOG were categorized into two categories, namely LR-FOG and LUR-FOG, depending on their reaction to levodopa, after receiving medication adjustments for at least 2 weeks.

Within the group of eligible patients with PD, we conducted three additional steps to specifically identify individuals with FOG. The identification of “off” and “on” states is determined by observing the response of motor and non-motor symptoms of PD to medication administration and their periodic recurrence. FOG is confirmed through by screening with the New Freezing of Gait Questionnaire (NFOG-Q) (Nieuwboer et al., 2009) score of 1 or higher, as well as clinical freezing events observed by two researchers (XW Chen and PY Wen) during activities such as 10-meter walking, turning, or passing through narrow doorways. Furthermore, FOG subtypes are identified by asking individuals about the specific circumstances in which they experience FOG. LUR-FOG PD patients experienced FOG independent of the “ON” or the “OFF” state of medication, whereas LR-FOG PD patients only experienced FOG at “OFF” state but not at the “ON” state, suggesting that freezing episodes can be relieved by dopaminergic medications.

Based on the previously mentioned criteria and protocols, the study included 71 PD patients in total, comprising 27 LR-FOG, 16 LUR-FOG, and 28 cases without FOG (NO-FOG). Nevertheless, two LR-FOG and one LUR-FOG patients were withdrawn from the research because of severe levodopa-induced motor fluctuations and discomfort during TMS assessment. Additionally, 22 HC who were matched for age, gender, and education level were selected. This study has been registered in the Chinese Clinical Trial Registry (ChiCTR2300076291). Every individual involved in the study gave their consent in writing after being fully informed.

### 2.2 Clinical assessment

All patients performed a range of clinical examinations and took part in semi-structured interviews while they were in the “on” condition. The assessment of motor and FOG-related functions was conducted using the Hoehn and Yahr (H&Y) staging, Unified Parkinson’s Disease Rating Scale part III (UPDRS-III), Tinetti Mobility Test (TMT), and NFOG-Q. Additionally, global cognition and executive function were assessed using the Mini-Mental State Examination (MMSE, >24) and Frontal Assessment Battery (FAB). The emotional state of the subjects was assessed utilizing the Hamilton Depression Rating Scale (HAMD) and the Hamilton Anxiety Rating Scale (HAMA). Furthermore, the total levodopa equivalent daily dose (LEDD) was computed to quantify the amount of dopaminergic medicine administered.

### 2.3 Electrophysiological assessment

#### 2.3.1 Resting motor threshold and input-output curve

To assess corticospinal excitability, we used a transcranial magnetic stimulator (YRD NS5000, Wuhan Yiruide Medical Equipment New Technology Co., Ltd.) with a maximum magnetic field strength of 3T, connected to a circular stimulating coil with a diameter of 10 cm for TMS. MEPs of the abductor pollicis brevis (APB) muscle was measured in PD patients on the affected side and in control subjects on their dominant side. Electromyographic signals were processed, amplified, filtered (bandwidth 1–2500 Hz), and then stored after being sampled at a rate greater than 10 kHz. Channels recording background muscle activity exceeding 100 mV within the first 200 ms before MEP onset were rejected.

When determining the hotspot of the APB, the coil’s intersection was aligned with the scalp tangential to the M1 area, with the coil handle pointing backward at a 45° angle from the midline. The coil was positioned over the M1 region to induce MEP in the APB, with the handle perpendicular to the midline and pointing toward the same side. We first examined the resting motor threshold (RMT), defined as the minimum output in which at least 5 out of 10 stimuli elicited MEP peak-to-peak amplitudes exceeding 50 μV. Subsequently, we measured recruitment curves (RC) with stimulus intensities ranging from 100 to 160% RMT in 10% increments. Five pulses were recorded at each stimulus intensity, and the average MEP for each set of stimulus intensities was calculated. Additionally, we determined the slope of the RC.

#### 2.3.2 SAI and LAI by paired associative stimulation

The method by which Tokimura et al. (2000), Sailer et al. (2003) described SAI and LAI was employed in our study. A MagProR30 magnetic stimulator system (Dantec Ltd., Denmark) with a maximum output of 2.0 T and an “8”-shaped figure-of-eight coil (MCF-B65) was used for TMS to primary motor cortex to induce MEP. Peripheral nerve stimulation and recording of electromyographic signals were carried out using an electromyography (Keypoint 9031A070, Dantec Ltd., Denmark). To evoke MEP, the “8”-shaped coil was placed at the optimal stimulation point of the motor cortex that controlled the contralateral APB muscle (2–3 cm next to the Cz point). The cortical magnetic stimulation intensity was set to induce MEPs in the relaxed APB, with a peak-to-peak amplitude of roughly 1 mV. Single pulses with a width of 200 ms were used as conditioning electrical stimuli, administered to the median nerve at the wrist (cathode proximal) using bipolar electrodes. A noticeable twitch in the relaxed APB muscle was elicited by setting the intensity of the conditioned peripheral stimulus just over the motor threshold. To record surface electromyographic signals from APB, participants were seated comfortably with both forearms slightly flexed and resting on a pillow, with the positive and negative electrodes placed on the motor spot of APB and the metacarpophalangeal joint of the thumb, respectively. The wrist joint’s middle was where the reference electrode was positioned. The electromyographic signals underwent amplification and band-pass filtering within the frequency range of 20 to 2,000 Hz.

**FIGURE 1:**
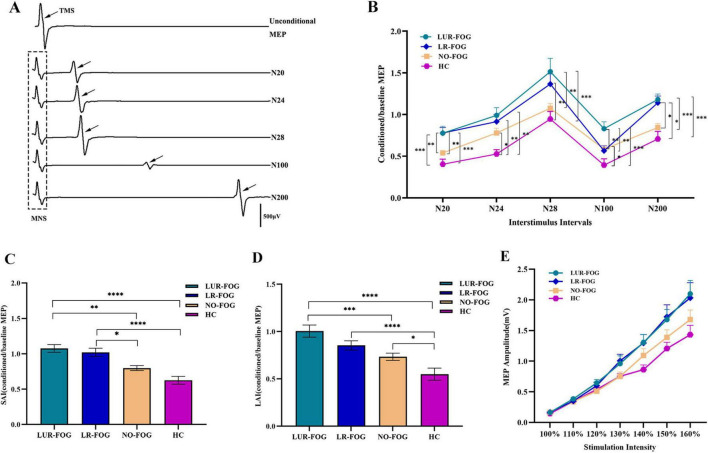
Comparison of SAI, LAI and input-output curves in the study participants. **(A)** A representative trace of afferent inhibition on cortical excitability examined by paired association stimulation recorded in control subjects (female, age 72), the waveform in the dotted box is the EMG induced by stimulation of the median nerve, and the waveform showed by the black arrow are the MEP generated by TMS. **(B)** Afferent inhibition tested by paired association stimulation at various ISI. At ISI 20 ms, in comparation with NO-FOG PD and HC, LUR-FOG PD patients showed lower SAI (increased MEP ratio) than LR-FOG PD patients. At ISI 24 ms, compared to HC, FOG PD and NO-FOG PD patients showed impaired SAI. At ISI 28 ms, compared to HC, both LR-FOG and LUR-FOG PD patients displayed decreased SAI; LUR-FOG PD had a more decreased SAI than NO-FOG PD patients. At ISI 100 ms, compared to LUR-FOG, NO-FOG, and HC groups, LR-FOG showed lower LAI; NO PD had a more reduced SAI than HC. At ISI 200 ms, compared to HC and NO-FOG, both LUR-FOG PD and LR-FOG patients exhibited lower LAI. **(C)** Comparison of SAI among the study groups. For the grand mean value of SAI, both subgroups of FOG-PD patients showed decreased SAI in comparison with HC as well as NO-FOG subjects. **(D)** Comparison of LAI among the study groups. For the grand mean value of LAI, compared to HC, LAI of NO-FOG decreased; while LAI of LUR-FOG was not only decreased compared to HC, but also reduced compared to NO-FOG PD patients. **(E)** Input-output curve of MEPs in patients with PD and HC. The seven stimulation intensities-100, 110, 120, 130, 140, 150, and 160% of RMT are displayed on the *x*-axis, while the *y*-axis displays the MEP amplitudes (mV). MNS, median nerve stimulation; TMS, transcranial magnetic stimulation. MEP: motor-evoked potential. ISI, interstimulus interval; HC, healthy control; NO-FOG, non-freezing of gait; LUR-FOG, levodopa-unresponsive freezing of gait; LR-FOG, levodopa-responsive freezing of gait; PD, Parkinson’s disease; SAI, short-latency afferent inhibition; LAI, long-latency afferent inhibition; Error bars indicate the standard deviation; *p < 0.05; **p < 0.01; ***p < 0.001; ****p < 0.0001.

Coupled peripheral electrical stimulation and cortical TMS were applied to evaluate SAI or LAI. Before cortical TMS, conditioning stimuli were administered to the median nerve at the wrist by a single electrical pulse at various interstimulus intervals (ISIs). The ISIs were set at 20, 24, 28 ms for SAI and 100, 200 ms for LAI (Figure 1A). The average amplitude of five consecutive MEP obtained from TMS on motor cortex alone (without conditioning stimulation), known as the baseline MEP, and the average amplitude of five consecutive MEP recorded from paired peripheral stimulation and cerebral stimulation, identified as the conditioned MEP, were computed for each ISI. In order to evaluate SAI and LAI, the conditioned MEP amplitude was calculated and reported as a percentage of the baseline MEP amplitude at each ISI. Electrophysiological assessments were performed on the dominant side in the control group and on the more impaired side in individuals with PD. During the experiment, all participants maintained complete relaxation with the aid of highly amplified electromyographic signals and visual feedback.

### 2.4 Gait assessment

Gait characteristics were obtained using a portable inertial measurement unit (IMU) system (GYENNO Science, China). Gait characteristics were assessed using 10-meter timed up and go (TUG) test (Criminger and Swank, 2020). In TUG test, participants were asked to rise from a seated position, ambulate a distance of 5 m, execute a 180° rotation, walk back, and then turn around again to sit down. It was instructed to the participants to walk at their self-selected comfortable speed. Each walking was completed twice, and the average value was taken. Prior to the formal test, each participant practiced twice. The gait characteristics of interest encompassed spatiotemporal parameters (Total TUG time[s], Gait speed [m/s], stride length [cm], Cadence [steps/min], Turning duration [s]).

### 2.5 Statistical analysis

The data analysis was performed utilizing SPSS 26.0 software. Gender differences were analyzed using the Chi-square test. The data’s normality was assessed through the use of histograms and the Shapiro-Wilk test. The Kruskal-Wallis test was used to assess the differences in age, education level, disease duration, H&Y staging, LEDD, MMSE, FAB, TMT scores, and recruitment curve slope or RMT tested by TMS because of the non-normal distribution of the data. The UPDRS-III scores for the three PD groups and spatial-temporal gait parameters were contrasted employing one-way analysis of variance (ANOVA). The NFOG-Q scores for the two FOG subtype groups were analyzed utilizing two-sample *t*-test. To increase normalcy, the log base-10 transformation was performed to the non-normally distributed gait characteristics, LAI and SAI variables. Repeated measures ANOVA was employed to compare the SAI variables obtained under various ISIs (with factor: ISIs 20, 24, and 28 ms) and different groups (between factor: LUR-FOG, LR-FOG, NO-FOG, and HC). Univariate ANOVA was utilized to compare the LAI variables obtained under different times (with factor: ISIs 100 and 200 ms) and different groups (between factor: HC, NO-FOG, LUR-FOG, and LR-FOG). To further lessen data variability, the grand mean of SAI was derived by averaging the SAI acquired at the ISIs 20, 24, and 28 ms (Manganelli et al., 2009), whereas the grand average of LAI was computed by averaging the ISIs 100 and 200 ms. One-way ANOVA was then used to evaluate the means of SAI and LAI. The differences in recruitment curve (RC) between the groups “NO-FOG PD, FOG PD (LR-FOG, LUR-FOG), and HC,” as well as the within-group factor “Stimulus intensity” (100, 110, 120, 130, 140, 150, and 160%) were compared employing repeated measures ANOVA. The significance level was set at *P* < 0.05, and *post hoc* analyses were carried out, when required, using Bonferroni adjustment for multiple comparisons when necessary. The associations between neurophysiological markers and gait parameters were investigated using Pearson correlation analysis, and multiple comparisons were corrected using False discovery rate (FDR) correction.

## 3 Results

### 3.1 Demographic and clinical data

The height, weight, education level, cognitive function, and emotional status were matched across each group. The FOG PD and NO-FOG PD exhibited comparable LEDD, H&Y stage, UPDRS-III. FOG PD patients had substantially poorer Tinetti balance, gait, and total scores in comparison with NO-FOG PD patients (Tinetti balance score: *P* < 0.0001; Tinetti gait score: *P* < 0.0001; Tinetti total score: *P* < 0.0001). However, there was no discernible variation in Tinetti scores between the two subgroups of individuals with FOG PD. The NFOG-Q scores of the LUR-FOG PD were considerably higher than that of LR-FOG patients (*P* = 0.014) (Table 1).

**TABLE 1:** Demographic, clinical evaluation in PD patients and HC.

	LR-FOG (*n* = 25)	LUR-FOG (*n* = 15)	NO-FOG (*n* = 28)	HC (*n* = 22)	*P*
Sex (M/F)	7/8	9/16	13/15	7/15	0.186^a^
Age (y)	65.56 ± 8.91	68.93 ± 7.14	65.76 ± 14.87	64.54 ± 9.23	0.682^c^
Weight (kg)	64.08 ± 9.43	60.23 ± 10.42	62.28 ± 11.49	60.89 ± 7.75	0.589^c^
Height (cm)	164.24 ± 9.04	161.07 ± 5.68	163.82 ± 7.79	162.09 ± 6.70	0.523^c^
Education (y)	9 (5, 12)	6 (3, 12)	9 (5, 12)	9 (4.75, 12)	0.921^b^
**Clinical data**					
Disease duration (y)	7 (6, 11)	5 (4,8. 5)	6 (3.25, 9)	NA	0.083^b^
LEDD (mg/d)	750 (600, 862.5)	550 (500, 750)	700 (475, 771.88)	NA	0.147^b^
MMSE	28 (26.5, 29)	28 (27, 29)	28.5 (25, 29.75)	NA	0.993^b^
HAMD	8 (4.5, 17.5)	11 (7, 11)	9 (6, 12.75)	NA	0.456^b^
HAMA	11 (6, 16)	8 (3, 11)	9 (5.25, 16)	NA	0.207^b^
FAB	17 (14, 18)	17 (16, 18)	17 (15, 17.75)	NA	0.436^b^
UPDRS-III	46 (37.5, 56)	36 (29, 48)	40 (26.25, 52.5)	NA	0.09^b^
H&Y stage	3 (2.5, 3)	3 (3, 3)	2.5 (2, 3)	NA	0.071^b^
Tinetti_balance	11 (9, 13)	10 (8, 13)	16 (15, 16)	NA	0.000^b^*
Tinetti_gait	7 (6, 9)	8 (7, 10)	11 (11, 12)	NA	0.000^b^*
Total Tinetti	18 (15, 22)	17 (14, 23)	27 (25.25, 28)	NA	0.000^b^*
NFOG-Q	20.92 ± 4.32	24.4 ± 4.10	NA	NA	0.014^d^*

HC, health controls; NO-FOG, non-freezing of gait; LUR-FOG, levodopa-unresponsive freezing of gait; LR-FOG, levodopa-responsive freezing of gait; M, male; F, female; y, years; NA, not applicable; LEDD, levodopa-equivalent daily dose; MMSE, Mini-Mental State Examination; HAMD, Hamilton Depression Rating Scale; HAMA, Hamilton Anxiety Rating Scale; FAB, Frontal Assessment Battery; UPDRS-III, Unified Parkinson’s Disease Rating Scale III; H&Y stage, Hoehn and Yahr stage; NFOG-Q, New Freezing of Gait Questionnaire;

*P < 0.05 was considered significant;

^a^χ^2^ test;

^b^Kruskal-Wallis test;

^c^analysis of covariance;

^d^Mann-Whitney *U* test.

### 3.2 Gait characteristics

Table 2 shows the spatiotemporal gait parameters of the four study groups. The total TUG time of FOG PD and NO-FOG patients was longer than of HC, with slower gait speed, shorter stride length, and longer turning duration (Bonferroni correction, all *P* < 0.05). For Cadence, there were no appreciable variations across the four groups. When comparing the LR-FOG FOG and LUR-FOG-FOG subgroups, no significant differences in gait characteristics was found.

**TABLE 2:** Descriptive spatiotemporal gait characteristics of all participants in TUG test.

	LR-FOG (*n* = 25)	LUR-FOG (*n* = 15)	NO-FOG (*n* = 28)	HC (*n* = 22)	*P*
Total TUG time (s)	37.68 ± 6.11	31.09 ± 3.99	22.06 ± 5.58	13.90 ± 2.08	0.000^c^*
Stride length (cm)	58.14 ± 21.02	68.29 ± 25.09	83.02 ± 20.49	109.97 ± 13.19	0.000^c^*
Gait speed (m/s)	0.54 ± 0.20	0.61 ± 0.23	0.76 ± 0.19	1.03 ± 0.14	0.000^c^*
Cadence (steps/min)	114.14 ± 17.28	108.96 ± 13.88	109.86 ± 6.32	111.85 ± 7.90	0.489
Turning duration (s)	5.82 ± 4.21	4.52 ± 3.11	2.62 ± 0.64	2.11 ± 0.38	0.006^c^*

HC, health controls; NO-FOG, non-freezing of gait; LUR-FOG, levodopa-unresponsive freezing of gait; LR-FOG, levodopa-responsive freezing of gait; TUG, timed up and go.

*P < 0.05 was significant;

^c^analysis of covariance.

### 3.3 Neurophysiological parameters

#### 3.3.1 Afferent inhibition

Figure 1B showed the comparison of SAI and LAI for FOG PD, NO-FOG PD and HC. For SAI variables at different ISIs, repeated ANOVA revealed meaningful main effects for GROUP (*F*_(3_,_86)_ = 9.94, *P* < 0.001) and TIME (*F*_(3_,_86)_ = 41.436, *P* < 0.001); no significant TIME × GROUP interaction was discovered. Specifically, at ISI 20 ms, in comparison to NO-FOG PD and HC, both LR-FOG and LUR-FOG PD patients exhibited decreased SAI (Bonferroni correction, both *P* < 0.001, Bonferroni-corrected). At ISI 24 ms, both FOG PD and NO-FOG PD patients showed impaired SAI in comparison to HC (Bonferroni correction, both *P* < 0.05). Both LR-FOG and LUR-FOG PD patients showed lower SAI at ISI 28 ms when contrasted with HC (Bonferroni correction, both *P* < 0.001); Additionally, LUR-FOG PD patients showed lower SAI than those with NO-FOG PD. When comparing LR-FOG and LUR-FOG subgroups, no significant differences in SAI were found. For LAI variables at different ISIs, ANOVA indicated significant main effects for GROUP (*F*_(3_,_86)_ = 8.839, *P* < 0.001) and TIME (*F*_(3_,_86)_ = 419.035, *P* < 0.001); no significant TIME × GROUP interaction was identified. Specifically, at ISI 100 ms, LUR-FOG PD exhibited reduced LAI compared to LR-FOG, NO-FOG, and HC groups (Bonferroni correction, all *P* < 0.05); however, LAI in NO-FOG PD was also reduced in comparison to HC. At ISI 200 ms, both LUR-FOG PD and LR-FOG PD patients showed reduced LAI in comparison to HC and NO-FOG (Bonferroni correction, all *P* < 0.05).

Compared to HC and NO-FOG participants, the grand mean of SAI for both FOG-PD subgroups was lower (Bonferroni correction, *P* < 0.001) (Figure 1C). In terms of the grand mean of LAI, LUR-FOG and LR-FOG PD patients had reduced LAI in comparison to HC (Bonferroni correction, *P* < 0.001), while decreased LAI in comparison to patients with NO-FOG was revealed for LUR-FOG (Bonferroni correction, *P* < 0.001), but not for LR-FOG (Figure 1D). No significant difference of the grand mean of SAI/LAI between LR-FOG and LUR-FOG was found.

#### 3.3.2 Resting motor threshold and input-output curves

RMT did not show discernible difference among PD patients and HC (*P* = 0.339). As anticipated, the analysis showed that stimulus intensity had a substantial impact on MEP amplitude [*F*_(3_,_86)_ = 194.738, *P* < 0.001], with an increase in MEP amplitude noted as stimulus intensity increased. Evaluation of input-output MEP curves demonstrated higher M1 excitability in PD patients compared to HC (Figure 1E), which was supported by a significant main effect of Group [*F*_(3_,_86)_ = 2.822, *P* = 0.044]. The interaction of stimulus intensity × Group was not statistically significant [*F*_(3_,_86)_ = 1.307, *P* = 0.183]. Additionally, RC slope did not differ among the four groups (*P* = 0.062).

### 3.4 Correlation analysis

**FIGURE 2:**
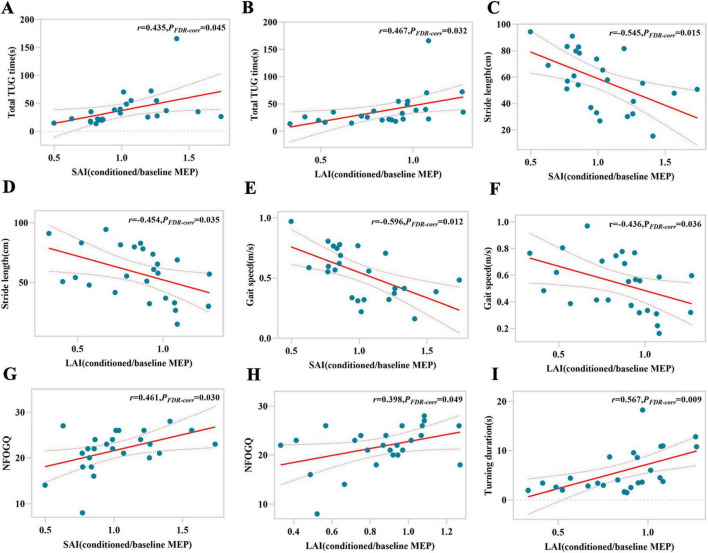
Correlations between gait characteristics and magnitude of SAI or LAI for LR-FOG PD patients. SAI, short-latency afferent inhibition; FDR-corr, false discovery rate correction; LAI, long-latency afferent inhibition; MEP, motor evoked potential; TUG, timed up and go; NFOG-Q, New Freezing of Gait Questionnaire.

**FIGURE 3:**
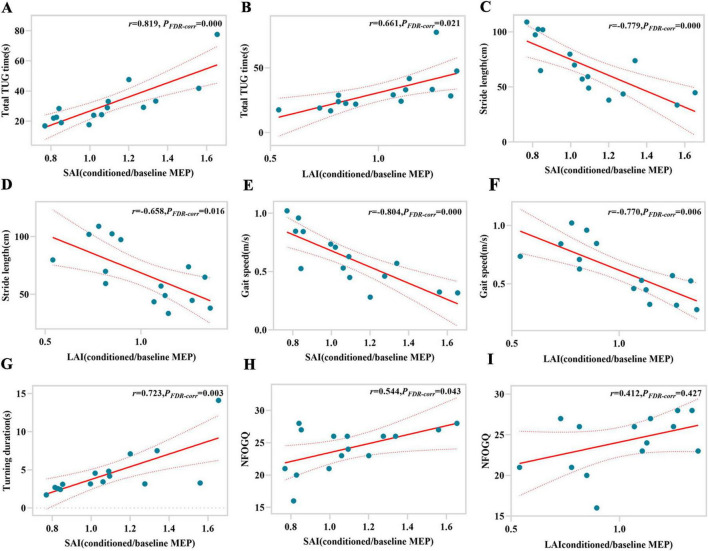
Correlations between gait characteristics and magnitude of SAI or LAI for LUR-FOG PD patients. SAI, short-latency afferent inhibition; FDR-corr, False discovery rate correction; LAI, long-latency afferent inhibition; MEP, motor evoked potential. TUG, timed up and go; NFOG-Q, New Freezing of Gait Questionnaire.

According to Pearson correlation analysis, for LR-FOG PD, SAI and LAI are not only significantly correlated with total TUG time (*r* = 0.435, *P*_*FDR–corr*_ = 0.045; *r* = 0.467, *P*_*FDR–corr*_ = 0.032), but also with stride length (*r* = −0.545, *P*_*FDR–corr*_ = 0.015; *r* = −0.454, *P*_*FDR–corr*_ = 0.035), gait speed (*r* = −0.596, *P*_*FDR–corr*_ = 0.012; *r* = −0.436, *P*_*FDR–corr*_ = 0.036), and NFOG-Q (*r* = 0.461, *P*_*FDR–corr*_ = 0.030; *r* = 0.398, *P*_*FDR–corr*_ = 0.049) (Figures 2A–H). Furthermore, there is a significant correlation between LAI and turning duration (*r* = 0.567, *P*_*FDR–corr*_ = 0.009) (Figure 2I). In the cases of LUR-FOG PD, there is a strong correlation between SAI and LAI with both total TUG time (*r* = 0.819, *P*_*FDR–corr*_ = 0.000; *r* = 0.661, *P*_*FDR–corr*_ = 0.021) and stride length (*r* = −0.779, *P*_*FDR–corr*_ = 0.000; *r* = −0.658, *P*_*FDR–corr*_ = 0.016), as well as gait speed (*r* = −0.804, *P*_*FDR–corr*_ = 0.000; *r* = −0.770, *P*_*FDR–corr*_ = 0.006) (Figures 3A–G). In addition, for LUR-FOG PD patients, there is a correlation between SAI and turning duration as well as NFOG-Q (*r* = 0.723, *P*_*FDR–corr*_ = 0.002; *r* = 0.544, *P*_*FDR–corr*_ = 0.043) (Figures 3G, H), but no correlation between LAI and NFOGQ was found (*r* = 0.412, *P*_*FDR–corr*_ = 0.427) (Figure 3I). No association of SAI/LAI with gait parameters were observed in patients with NO-FOG PD or HC.

## 4 Discussion

This study explored the alterations of afferent inhibition and analyzed the associations of SAI/LAI magnitude with the gait performance in two subgroups of PD with FOG (LR-FOG and LUR-FOG). Our results indicated that SAI is remarkably reduced in both LUR-FOG and LR-FOG PD patients in relative to NO-FOG PD or HC, but not significantly different between the two PD-FOG subgroups. LAI was also decreased in the two PD-FOG subgroups in comparison to HC, while only LUR-FOG, not LR-FOG PD, showed reduced LAI in comparison to NO-FOG PD. In TUG task, both subgroups of PD-FOG exhibited poorer gait performance with deteriorated spatiotemporal parameters compared to HC or NO-FOG PD patients. Furthermore, in the analysis of the correlations between SAI/LAI and gait spatiotemporal parameters, it was found that SAI and LAI were correlated with total TUG time, stride length, gait speed, and NFOGQ. Taken together, the reduction of SAI or LAI in PD patients with FOG were correlated to disturbed gait performance and the scores of NFOGQ, indicating that sensorimotor integration dysfunction contributes to the development of FOG in PD patients.

SAI reflected the fast-inhibitory impact of a conditioning peripheral electrical stimulus on the motor response triggered by a TMS test pulse delivered to the contralateral primary motor cortex (M1). Although, theoretically, dysfunction in either afferent pathway, sensorimotor cortex or fast corticomotor output pathway may lead to abnormality of SAI, the lack of significant damage to the fast sensory-cortical pathway and the pyramidal tract pathway in PD suggests that SAI abnormalities in PD are the result of impaired functionality in rapid sensory-motor integration within the cortex (Dubbioso et al., 2019). Our study showed that SAI was reduced in LR-FOG as well as LUR-FOG patients in comparison with HC and NO-FOG PD. Additionally, we observed that SAI was positively correlated with total TUG time and NFOGQ score, and negatively correlated with stride length and gait speed. These findings suggested that reduced SAI was linked to compromised gait performance in PD with FOG, consistent with the results of previous studies by Rochester et al. (2012) and Pelosin et al. (2016), supporting the view that fast cortical sensory-motor integration dysfunction is involved in the development of FOG in PD. However, it should be noted that the changes of SAI in PD were variable in the published reports, ranging from decreased to normal or even elevated SAI findings. In addition, alteration of SAI and its relation to gait disturbances in PD were neither consistent in previous researches; One research conducted by Picillo et al. (2015) on PD with FOG, no evidence was found to support alteration of SAI in this subtype of patients. The variability can be due to the influence of various factors on the strength of SAI, such as clinical phenotype of PD, differences in dopaminergic treatment and the magnitude of dopaminergic or cholinergic neurodegeneration. Indeed, reduced SAI has been reported in PD patients with more severe motor symptoms in the “on medication” state, impaired cognition function, and symptoms related to a higher risk of cognitive deterioration, which include visual hallucinations (Manganelli et al., 2009), olfactory dysfunction (Oh et al., 2017; Versace et al., 2017) and REM-sleep Behavior Disorders (Nardone et al., 2013). In the present study, the disease duration, scores of H/Y stage, UPDRS-III, MMSE, HAMD and HAMA and LEDD showed no significant differences between FOG PD and NO-FOG patients, implying that factors that may affect SAI, such as dopaminergic medication, cognitive function, severity of motor symptoms and disease duration and stages, were not be the main reason that lead to the weakened SAI in PD with FOG in relative to NO-FOG patients. Nevertheless, the modulative effects of dopaminergic medication on SAI cannot be ruled out in the PD patients in our study, because the anti-PD drug regimen was different despite comparable LEDD for PD patients in the three groups. An interesting result in our study was that PD without FOG showed decreased SAI in relative to HC only at ISI of 24 ms, but not at other ISI despite impaired gait in these patients, indicating that SAI at various ISI may have different sensitivity in probing the alteration of rapid sensorimotor interaction in cortical level.

The mechanism of reduced SAI in PD with FOG was not clear. As mentioned above, the aberrant SAI in PD may well be attributed to the impairment in fast intracortical sensorimotor integration, however, the exact circuit underlying this interaction is still unknown. Despite not directly receiving sensory information, the basal ganglia play a crucial role in sensorimotor integration. The malfunctioning of the basal ganglia in PD may have a significant impact on sensory-motor interaction in the cortex. Studies in PD patients receiving deep brain stimulation of subthalamic nuclear (STN-DBS) showed that reduced SAI could be restored by acute STN stimulation (Sailer et al., 2007) or chronic STN-DBS (Wagle Shukla et al., 2013), demonstrating that disturbed basal ganglia output to the sensorimotor cortex was involved in development of the abnormal SAI in PD.

Increasing clinical and pharmacological evidences suggest that neurotransmitters including acetylcholine, dopamine, and GABA modulate the expression of SAI (Turco et al., 2018). It is well established that SAI was closely influenced by central cholinergic system. SAI deficiency has been demonstrated in people with cholinergic dysfunction, specifically in those with AD and PD accompanied by mild cognitive impairment (Schirinzi et al., 2018). SAI has historically been thought of as a stand-in for cholinergic activity in the brain. It can track functional degradation of central cholinergic circuits to identify cholinergic dementia from non-cholinergic dementia (Di Lazzaro et al., 2006; Manganelli et al., 2014; Dubbioso et al., 2017). Presently, there is a belief that the decrease in SAI can be attributed, at least in part, to the simultaneous presence of cholinergic impairments at the cortical level (Dubbioso et al., 2019). Interestingly, FOG in PD patients has also been linked to impaired cognition function and degeneration or dysfunction of central cholinergic neurons (Bohnen and Albin, 2011), especially in nucleus basalis of Meynert (NBM) in the basal forebrain (Pasquini et al., 2021; Dalrymple et al., 2021; Bohnen et al., 2014) and the cholinergic neurons of PPN in the upper brainstem (Bharti et al., 2019; Morris et al., 2019; Windels et al., 2015; Wang et al., 2016). Thus, the impairment of cholinergic activity in the NBM-cortex and PPN-thalamus pathways may play a part in the complex pathophysiological mechanism of FOG and is associated to the reduced SAI in PD with FOG. In addition to cholinergic system, dopamine system is also involved in the regulation of SAI. However, the relation of dopamine with SAI was complex. On one hand, some studied found that SAI was not affected (Degardin et al., 2012; Zamir et al., 2012; Picillo et al., 2015; Nelson et al., 2018), or even enhanced (Di Lazzaro et al., 2004; Nardone et al., 2005) in PD patients without receiving dopaminergic treatment; on the other hand, decreased SAI was consistently observed in the ON-medication state (Martin-Rodriguez and Mir, 2020), indicating that dopamine replacement may be responsible for this anomaly. An intriguing hypothesis might be that a decrease thalamo-cortical drive triggered by nigrostriatal dopaminergic denervation may lead to increase in SAI, an impact that could be hidden by coexistence of cortical cholinergic deficits, and chronic dopamine replacing therapy may correct the enhanced SAI and thus give prominence to the action of impaired cholinergic system. This theory of dopaminergic regulation of SAI warrants further study. Studies suggest that GABA_A_ receptor agonists (benzodiazepines) can decrease SAI (Di Lazzaro et al., 2007; Di Lazzaro et al., 2005a; Di Lazzaro et al., 2005b). Given that GABA is known to regulate acetylcholine release in both brainstem and cortical regions (Giorgetti et al., 2000), SAI may involve a cholinergic pathway modulated by GABAergic activity. In summation, dysfunction of the cholinergic, dopaminergic or GABAergic systems, or the dysregulated interaction between these neurotransmitter systems, may lead to the reduced SAI in FOG patients.

Our study also found that LAI was decreased in PD with FOG patients, but with some difference between LR-FOG PD and LUR-FOG PD. Specifically, LAI (grand mean, at ISI 100 and 200 ms) in LUR-FOG was significantly reduced in comparison to NO-FOG PD or HC; Moreover, LUR-FOG PD exhibited reduced LAI at ISI 100 ms (not for LAI at ISI 200 ms or the grand mean of LAI) compared to LR-FOG. For LR-FOG PD, LAI was also reduced in comparison to HC, but only LAI at ISI 200 ms, not LAI at ISI 100 ms or grand mean of LAI was decreased in relative to NO-FOG. Furthermore, we also observed that there was a positive relationship between LAI and total TUG time or NFOGQ scores (for LR-FOG group), and a negative relationship between LAI and stride length, as well as gait speed in PD-FOG patients. These findings provided further evidence that dysfunction of sensorimotor integration was engaged in the occurrence of FOG with PD. As far as we are aware, this study was the first to investigate alterations of LAI and its association with gait impairments in different FOG subtypes of PD patients.

The neural substrate and mechanism for LAI are poorly understood. Given the relatively long intervals between peripheral electrical stimulation and TMS pulses, there is a possibility for widespread activation of various somatosensory cortical regions that react to incoming sensory inputs (Turco et al., 2018). Studies have demonstrated that primary somatosensory cortex (S1), posterior parietal cortex (PPC), and secondary somatosensory cortex (S2) are activated within the initial 200 ms following peripheral stimulation (Allison et al., 1989; Allison et al., 1992), and these cortical areas project to M1, potentially participating in mediating inhibition within M1 (Turco et al., 2018). LAI may also involve the basal ganglia thalamocortical loop (Sailer et al., 2002; Chen et al., 1999; Sailer et al., 2003; Abbruzzese et al., 2001), Sailer et al. (2003) found a decrease or absence of LAI in PD and speculated that abnormal processing of incoming sensory information in the basal ganglia region, which functions as a sensory analyzer, might contribute to the motor manifestations of PD (Brown et al., 1997). Current research has discovered the typical modulation of LAI connected to mobility (Richardson et al., 2008; Pirio Richardson et al., 2009) but it still remains unclear whether alterations in LAI are associated with any specific symptoms or indicators of PD. Our study results indicated that reduced LAI may be associated with FOG for both levodopa responsive and unresponsive subtypes. It has been suggested that abnormalities in LAI may lead to difficulty in determining joint positions while moving, thus resulting in scaling errors in movement amplitudes in PD (Berardelli et al., 1986). The networks involved in gait control encompass several brain regions, such as the basal ganglia, sensorimotor cortex, prefrontal cortex, post parietal areas and upper brainstem (especially the PPN). Hence, we speculated that the reduction in LAI may be related to damage to the basal ganglia-thalamocortical loop or impaired integration of sensorimotor areas including primary and secondary somatosensory cortex, PPC, and motor and premotor cortex. Interestingly, our study revealed that LAI was reduced in both LR-FOG and LUR-FOG PD groups, but with some distinctions. Specifically, only LAI in LUR-FOG, but not in LR-FOG, was significantly reduced in contrast to NO-FOG, and LUR-FOG PD exhibited reduced LAI at ISI 100 ms compared to LR-FOG, indicating that weakening of LAI in LUR-FOG seemed to be more significant than LR-FOG. Since reduced LAI in PD and gait disturbance in LUR-FOG were not affected by dopaminergic medication, we speculated that the decrease in LAI in LUR-FOG may be related to the dysfunction of non-dopaminergic transmitter system such as cholinergic system, rather than dopaminergic loss. These findings may guide the development of methods to improve FOG, such as using physical therapy enhancing proprioception input (e.g., vibration stimulation) to improve gait performance in PD patients with poor response to levodopa treatment.

It should be acknowledged that our study has certain limitations. Firstly, only five trials were collected for each condition. Nevertheless, five trials for each condition were also be used by some investigators. Previous studies, using the same technical procedure as our study, have succeeded in finding a reduced SAI in patients with visual hallucinations (Manganelli et al., 2009) and in PD patients with LUR-FOG (Wang et al., 2022). Secondly, our sample size is small, and augmenting it in subsequent investigations could bolster the dependability of our findings. Existing studies indicated that dopaminergic medications may result in decreased central processing or integration of sensory signals in PD patients (Wagle Shukla et al., 2013). Additionally, our study was carried out in the “ON” state of medication. We cannot completely eliminate influence of medication on our results. Nevertheless, variations in LEDD among the patients in our study are not remarkable, suggesting that the drug had a minor effect on the experimental outcomes. Moreover, the categorization of FOG is based on subjective historical information without any objective indications. Future study should aim to provide more clarity on the criteria for classification and explore the use of more objective standards in classifying FOG. Finally, while this study examined how the sensorimotor integration function contributes to PD-FOG from a neurophysiological standpoint, additional imaging investigations are required to clarify the linkages within the functional brain network and increase the confidence in our findings.

## 5 Conclusion

In conclusion, SAI or LAI in PD with FOG was reduced, with LAI being more significantly weakened in LUR-FOG in relative to LR-FOG and the scores of NFOGQ, and the magnitude of reduction of SAI/LAI was associated with impaired gait performance, indicating that sensorimotor integration dysfunction played a role the development of FOG in PD patients.

## Data Availability

The raw data supporting the conclusions of this article will be made available by the authors, without undue reservation.
